# Determinants of the Cancer Drug Funding Process in Canada

**DOI:** 10.3390/curroncol29030162

**Published:** 2022-03-15

**Authors:** Joanna Gotfrit, Ashley Jackson, John J. W. Shin, David J. Stewart, Ranjeeta Mallick, Paul Wheatley-Price

**Affiliations:** 1 Department of Medicine, University of Ottawa, Ottawa, ON K1H 8L6, Canada; dstewart@toh.ca (D.J.S.); pwheatleyprice@toh.ca (P.W.-P.); 2 Ottawa Hospital Research Institute, Ottawa, ON K1Y 4E9, Canada; ajack028@uottawa.ca (A.J.); jshin059@uottawa.ca (J.J.W.S.); rmallick@ohri.ca (R.M.)

**Keywords:** drug funding, cancer drugs, drug access

## Abstract

Background: Canada has a publicly funded healthcare system with a complex drug funding process. After Health Canada approval to market a drug, the pan-Canadian Oncology Drug Review (pCODR) (now renamed the CADTH reimbursement review) makes a non-binding funding recommendation to the Canadian provinces (except Quebec), which each then decide whether the drug will be publicly funded. We identified the determinants of funding in this process. Methods: We analyzed drugs for advanced lung (*n* = 15), breast (*n* = 8), colorectal (CRC) (*n* = 7), melanoma (*n* = 10), and neuroendocrine (NET) (*n* = 3) cancers undergoing the funding decision process from 2011 to 2019. Determinants of funding assessed in the model included list price, cancer type, drug class, and pCODR recommendation. The primary outcome was the correlation between list price and time to funding (TTF: Health Canada approval to first provincial funding). Secondary outcomes included an exploratory analysis of predictors of drug funding. Results: We analyzed 43 drugs: targeted agents 72%, immunotherapy 20%, chemotherapy 7%. A total of 72% were funded in at least one province. Median TTF was 379 days (IQR 203–601). Median list price (28-day course) was CAD 8213 (IQR CAD 5391–9445). Higher list price was not correlated with TTF (correlation coefficient −0.20, *p* = 0.28). There was no association between list price and pCODR recommendation or the decision to fund in at least one province. A positive pCODR recommendation correlated with the provinces’ funding decisions (*p* < 0.001), where 89% of drugs with a positive recommendation were funded and 100% of drugs with a negative recommendation were not funded. Tumor type was predictive of TTF (*p* < 0.001): CRC drugs were the slowest at a median of 2541 days (IQR 702–4379), and NETs were the quickest at a median of 0 days (IQR 0–502). Cancer type predicted decision to fund in at least one province (*p* = 0.005), with funding for 100% of NET drugs at the high end and 29% of CRC drugs at the low end. Drug class was predictive of TTF (*p* = 0.01): 465 days (IQR 245–702) for targeted agents, 443 days (IQR 298–587) for chemotherapy, and 339 days (IQR 164–446) for immunotherapy. Conclusions: Determinants of drug funding included cancer type, drug class, and pCODR recommendation but not list price. Factors other than cost were more heavily weighted in the funding decisions of cancer drugs in Canadian provinces.

## 1. Introduction

Cancer is the leading cause of death in Canada, with patients often diagnosed with advanced disease [1]. The development of new cancer drugs with novel mechanisms of action has contributed to improved survival rates in the setting of advanced malignancy [2]. However, the cancer drug regulatory and funding process in Canada is complex and may delay access. Once a drug demonstrates proof of efficacy (POE) in clinical studies (usually, but not exclusively, phase III clinical trials), the drug must receive Health Canada approval in order to be marketed in Canada [3,4]. With Health Canada approval, cancer drugs can be sold in the country, but their cost is not by default reimbursed by provincial and territorial payors. In order to receive public funding, cancer drugs must undergo a Health Technology Assessment (HTA) by the CADTH pan-Canadian Oncology Drug Review (pCODR) (since data collection for this manuscript it has been re-named the CADTH Reimbursement Review) [5]. The goal of the pCODR HTA is to make a legally non-binding, evidence-based funding recommendation to the provinces and territories of Canada (except Quebec, where the Institut National d’Excellence en Sante et en Services Sociaux (INESSS) undertakes a different process). The pCODR recommendation is based on clinical benefit, patient values, cost effectiveness, and feasibility of adoption into health systems. For each drug submitted for pCODR review, a recommendation is made to either fund the drug, not fund, or fund conditionally [6]. With a positive recommendation, a confidential price negotiation can occur with the pan-Canadian Pharmaceutical Alliance (pCPA). When a price is agreed upon, the HTA recommendation can then be considered by the provinces and territories individually to determine whether or not a given drug will be publicly reimbursed.

The complexity of this process may inadvertently restrict or delay access to new, life-prolonging medicines. It has been shown that the median time to public funding for cancer drugs in Canada is over 26 months from the date of proven drug efficacy. As a result, during the regulatory and funding process of 21 drugs used in advanced lung, breast, and colorectal cancer in Canada, over 39,000 potential life years were lost while awaiting public funding [3]. Similarly, in a study of 27 drugs used for advanced cancer, it was estimated that for each year eliminated from the regulatory and funding process, a median of 79,920 life years would be saved worldwide [7].

While it is known that the provincial and territorial payers base their funding decisions on the pCODR review, jurisdictional priorities, budget impacts, and program mandates [8], the factors most associated with funding decisions and timeliness are unknown [9,10,11]. To better understand the factors associated with positive funding decisions and timeliness, we investigated the determinants of the cancer drug funding process in Canada including the pCODR recommendation, list price, tumor type, and agent class.

## 2. Methods

We analyzed 43 drugs for advanced breast cancer, colorectal cancer (CRC), lung cancer melanoma, and neuroendocrine tumors (NETs) undergoing the funding decision process in Canada between 2011 and 2019.

The local research ethics board was consulted and determined that ethics approval was not required for this study because all data points collected and analyzed were publicly available.

### 2.1. Endpoints

The primary endpoint was the correlation between the publicly stated list price and time to funding (TTF) among the drugs that were funded in at least one province. TTF was defined as the time from Health Canada approval (Notice of Compliance (NOC)) to first decision to fund by a province or territory. List prices were standardized as the price for a 28-day course.

Secondary endpoints included an exploratory analysis of predictors of both the decision to fund as well as funding timeliness, including the pCODR recommendation, tumor type, agent class, and list price.

### 2.2. Eligibility Criteria

Drugs that were eligible for inclusion were all drugs used in the treatment of advanced breast cancer, CRC, lung cancer, melanoma, and NETs submitted to the pCODR HTA from 2011 to 2019. Data points for all included drugs were updated and current as of April 2019. Drugs still under provincial consideration were considered not funded for the purposes of the analysis. The time point of first public funding was set as the date on which the first province or territory agreed to publicly reimburse the drug. Drugs without NOC (approval by Health Canada) were excluded from the analysis due to missing essential data points (such as list price).

### 2.3. Calculations

When determining the drug cost per 28-day course, additional costs for loading doses in cycle 1 were excluded from the calculations. In the case of Ipilimumab, for the indication in the study (four courses only), the cost of the loading dose was included in the calculation. The cost of each drug was calculated separately with the exception of drugs submitted as doublets to pCODR, which were calculated as the cost for both drugs for a 28-day course.

When calculating median list price, all drugs in the dataset were included regardless of funding status.

To calculate the median TTF, the median time from Health Canada approval to first provincial or territorial decision to fund was determined. Only drugs that received funding in at least one province or territory were included in this calculation.

### 2.4. Statistics

Statistics were primarily descriptive due to the small sample size. The continuous variables were summarized using median (IQR) and compared using the Wilcoxon rank-sum test. Categorical variables were summarized using frequency (%) and compared using the chi-square test. Calculations of correlation coefficients were performed where applicable, and *p* < 0.05 was considered significant.

A logistic regression model was constructed for the outcome of decision to fund, using as predictors cost per 28-day course and agent class. The results are presented as odds ratios (95% CI). pCODR recommendation was not included in logistic regression as all drugs that received a “do not fund” recommendation were not funded, and the model required at least one instance of each possible outcome. The tumor type was not included in logistic regression due to the small sample size and five levels of the variable.

## 3. Results

We analyzed 43 cancer drugs that underwent the funding decision process between 2011 and 2019. These included drugs for breast cancer (*n* = 8), CRC (*n* = 7), lung cancer (*n* = 15), melanoma (*n* = 10), and NETs (*n* = 3). See Table 1 for the list of included drugs and descriptors. Of the included drugs, 3 were cytotoxic chemotherapies, 9 were immunotherapies, and 31 were targeted therapies. Among the 43 drugs observed, 35 received a favorable pCODR recommendation (to fund or fund conditionally), and 31 were funded in at least one province. The TTF ranged from 0 to 4379 days, with a median of 379 days. The list price ranged from CAD 1166 to CAD 34,305 for a 28-day course with a median list price of CAD 8213. Table 2 summarizes the study drug characteristics.

### 3.1. Determinants of Time to Funding (TTF)

**Figure 1:**
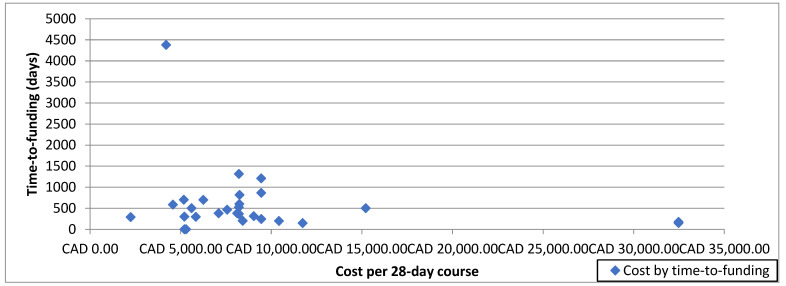
Scatter plot of time-to-funding by cost per 28-day course.

There was no correlation between list price and TTF (correlation coefficient −0.20, *p* = 0.28) (Figure 1). However, there were significant differences in TTF based on drug characteristics. Among the different tumor types, drugs for NETs had the shortest median TTF of 0 days (IQR 0–502), while drugs to treat CRC had the longest median TTF at 2541 days (IQR 702–4379) (*p* < 0.001). Among different classes of drugs, immunotherapies had the shortest median TTF at 339 days (IQR 164–446), while targeted therapies had the longest median TTF of 465 days (IQR 245–702) (*p* = 0.01). See Table 3 for the TTF based on drug characteristics.

### 3.2. Determinants of Provincial/Territorial Funding Decisions

The pCODR recommendation was significantly associated with the provincial/territorial funding decisions (*p* < 0.001). Of the 35 drugs that received a positive pCODR recommendation, 31 (89%) were funded in at least one province by the time of data cutoff. Of those funded in at least 1 province (*n* = 31), 4 (13%) were funded in 1–5 provinces, and 27 (87%) were funded in 6 or more provinces. Of the eight drugs that received a negative pCODR recommendation, none have been funded in any province (0% uptake). 

**Figure 2:**
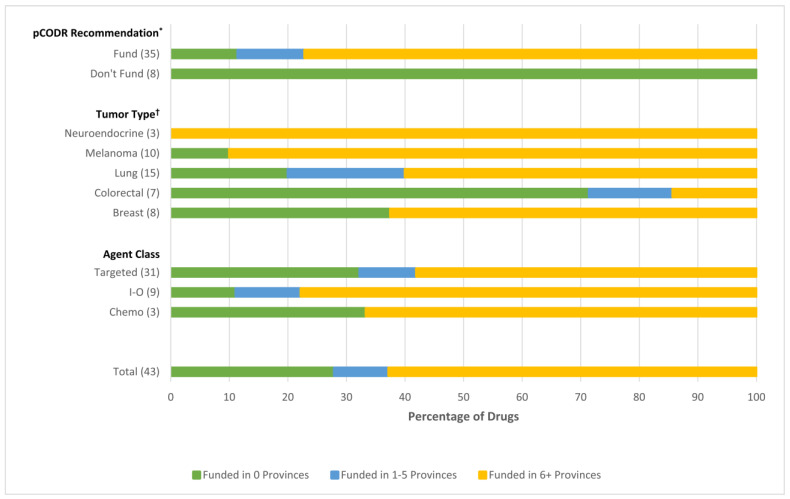
Determinants of drug funding * *p* < 0.0001, ^†^ *p* = 0.0347. Abbreviations: I-O, immunotherapy.

Tumor type predicted the decision to fund, with funding in at least one province for 100% of NET drugs, 90% of melanoma drugs, 80% of lung cancer drugs, 63% of breast cancer drugs, and 29% of CRC drugs (*p* = 0.005) (Figure 2).

There was no statistically significant difference in drug funding by drug class, with funding in at least one province for 89% of immunotherapies, 68% of targeted therapies, and 67% of chemotherapies (*p* = 0.52). In comparison to immunotherapy drugs, the odds ratio (OR) for the decision to fund chemotherapy drugs was 0.11 (95% CI 0.002–6.48), and for targeted drugs was 0.12 (95% CI 0.005–3.09). Statistical significance could not be established due to the small sample size.

### 3.3. Association of Drug Characteristics and List Price

There were significant differences in list price (for a 28-day course) among drugs for the different tumor types. Melanoma drugs cost the most with a median list price of CAD 9319 (IQR CAD 8120–32,480) while breast cancer drugs had the lowest median list price of CAD 5729 (IQR CAD 4259–7334) (Table 4). Per CAD 500 decrease in list price, the OR for the decision to fund was 1.03 (95% CI 0.96–1.11), and per CAD 1000 decrease in list price, the OR for the decision to fund was 1.06 (95% CI 0.92–1.22). With regard to agent class, immunotherapy had the most expensive list price with a median of CAD 9305 (IQR CAD 8213–32,480), while chemotherapy had the lowest list price at a median of CAD 5631 (IQR CAD 4569–5834). There was no association between list price and the pCODR recommendation or the decision to fund in at least one province (Table 3).

## 4. Discussion

The drug regulatory and funding process in Canada is complex and may result in delays in public access to life-prolonging anticancer therapies. While clinicians prescribe medications based on clinical judgement, risks, and benefits, the regulatory approval and funding decisions of cancer drugs in Canadian provinces are ultimately political processes. The specific factors correlated with positive provincial funding decisions and timeliness have been minimally investigated. We examined factors potentially influencing the drug regulatory and funding process including drug price, pCODR recommendation, tumor type, and agent class, to better understand the determinants of cancer drug funding decisions in Canada.

### 4.1. Impact of Drug Price

While our study showed that the cost of cancer drugs varies widely, it negated the intuitive assumption that the list price of anticancer therapies significantly influences regulatory and funding decisions or their timeliness. In fact, our study suggested no correlation between the list price and the speed with which cancer drugs are funded, and logistic regression did not reveal increased odds of funding due to decreased price. Similarly, there were no significant differences among list price between drugs that received positive or negative pCODR recommendations or between those that did or did not ultimately receive funding in at least one province. These findings may suggest that cost is not the most important driving factor in the drug funding process in Canada. It should be noted though that the publicly available list price does not represent the final price paid by the provinces. This is confidentially negotiated at pCPA prior to provincial listings.

While our study found no association between list price and funding, it is clear that the economic burden of cancer in Canada is a growing area of concern. One study found that between 2005 and 2012, the average annual economic burden of cancer care in Canada increased from CAD 2.9 to 7.5 billion [12]. A 2018 study that surveyed policymakers with cancer drug funding decision-making experience across nine Canadian provinces found that the most common reason cited for a drug to not receive funding despite a positive pCODR recommendation was budget constraints, that said, the majority (64%) of policymakers also cited that negative funding decisions may be made if the drug in question is not a priority for the local tumor group [13]. This may indicate that drug- and cancer-specific factors may play a larger role. Therefore, drugs addressing an unmet need may be considered at the provincial level differently to those where an existing drug in the same class is already available.

Assessing the role of drug cost in drug funding decisions and timeliness may be an over simplification, as cost in isolation makes no comment on the value of the drug in question. Drugs may be better analyzed by their cost effectiveness, which reports the cost of a drug adjusted by its clinical efficacy (for example: the cost per life year gained). A study of cancer care funding decisions in British Columbia up to 2008 [14] showed that there was a negative association between the incremental cost-effectiveness ratio (ICER) per life year gained and the likelihood of receiving a positive funding recommendation. Furthermore, the study found a significant difference between the mean ICER per life year gained among cancer drugs that received positive reimbursement recommendations (CAD 42,006) and those that received negative recommendations (CAD 156,967), indicating that drugs with better cost effectiveness were associated with positive funding recommendations. It would, therefore, seem that raw cost may not be as meaningful in decision-making as cost relative to drug efficacy. Focus groups have identified several factors that the Canadian public considers important when determining cost effectiveness of cancer drugs, including improvement in quality of life, duration of life, restoring patient independence, and improving patient mental health [15].

### 4.2. Impact of pCODR Recommendation

CADTH/pCODR is responsible for advising the provinces of Canada on cancer drug funding decisions. Clearly, the pCODR recommendation influences provincial funding decisions, particularly when negative, as no drugs in our study with a negative pCODR recommendation were funded in any province. A negative HTA recommendation generally indicates that price negotiations with pCPA will not occur, thereby inhibiting provinces from pursuing funding. pCODR recommendations are based on an assessment of clinical benefit, economics, and patient values [6]. However, there are no publicly stated, transparent weighting schemes or objective thresholds for these criteria [9]. The influences contributing to the pCODR drug funding recommendations are vitally important. Analyses have suggested that a high quality of clinical evidence and a maximum incremental cost effectiveness ratio (ICER) of approximately CAD 140,000 per quality adjusted life year (QALY) are factors related to positive pCODR recommendations [16]. Interestingly, recently, CADTH/pCODR recommended the funding of adjuvant osimertinib in NSCLC if the cost effectiveness can be improved to CAD 50,000 per QALY, at this time necessitating a price reduction of 82% from the current list price [17]. The implications of CADTH making recommendations on pricing, which is also addressed by both the PMPRB and pCPA, is uncertain but, at the time of writing, is adding some degree of confusion to understanding the process.

Given the importance and necessity of the pCODR recommendation prior to provincial funding decisions, the rapidity of the pCODR assessment is critical to timely drug access. While the pCODR does have publicly stated timeline targets to produce their recommendations, these targets are met only 50% of the time [18]. It is clear that pCODR recommendations play a significant role in drug funding decisions in Canada. Thus, timeliness of the pCODR process is necessary for prompt funding decisions and access to potentially life-prolonging cancer drugs. That being said, the HTA process does not represent the longest time delay in approval of new drugs. This can come from earlier in the process, with a delay in applying for Health Canada approval or submission to the HTA process after positive clinical trials, or later in the process, with delays in negotiating the final price to be paid by provinces. A previous review suggested the median time from HTA completion to first public funding is 6.9 months but may be as long as over 2 years [3].

### 4.3. Impact of Tumor Type

We found that there were significant differences in the decision to fund as well as TTF among drugs used to treat different tumor types. Drugs that treat NETs were frequently funded and had the quickest TTF. However, it should be noted that there was a very small sample size of NET drugs in our study, and two of the three drugs used to treat NETs received funding in British Columbia before Health Canada approval was obtained, which is a rare circumstance and, therefore, may not accurately reflect the average TTF among drugs in this category. After NETs, breast cancer drugs were funded the quickest. On the other end of the spectrum, drugs used to treat CRC were infrequently funded and, when funded, took the longest. In fact, bevacizumab for colorectal cancer had the longest TTF, largely due to a long interval between Health Canada approval and submission to the HTA [3]. The differences in the decisions to fund and TTF by tumor type suggest that the type of cancer may influence funding decisions and urgency. The specific reasons for variable funding and timeliness by tumor type are beyond the scope of this analysis; however, we may hypothesize that various factors may be contributing such as disease prevalence, mortality, other treatments available, and clinical benefit of the drugs. Comparing the analyzed breast cancer and colorectal cancer drugs in our study, a greater proportion of the breast cancer drugs were used in the first line, and the range of PFS and OS gains were objectively more impressive (data not shown), which may account for some differences.

Additionally, it is possible that variable drug funding decisions and timelines by tumor type are related to politics. As cancer drug funding is a highly politicized issue, studies have investigated the role of media in drug funding decisions. One study found that increased media attention toward a drug corresponded with a quicker TTF, for example, for the drug trastuzumab used in breast cancer [19]. Factors cited as potential reasons for the unusually rapid funding of trastuzumab included media coverage and survivorship [19]. Breast cancer is a highly publicized disease [20,21,22,23,24]. We may hypothesize that the strong media presence around breast cancer may contribute to political pressure to fund breast cancer drugs more quickly than therapeutics for other tumor types that are less prevalent in modern media. In fact, political pressure, in the form of advertisements or from pharmaceutical companies, has been cited as a top reason for funding drugs that received a negative pCODR recommendation [13], although this would seem to occur rarely, and there are no examples of this among the drugs included in our study. Therefore, it is possible that these same political pressures influence the timeliness of funding decisions in Canada. While pressures originating in the media may potentially influence drug funding decisions and timeliness, there is no evidence that drug funding decisions are influenced by upcoming political elections. Studies have failed to show a significant difference in drug funding decisions in the 60-day period preceding provincial elections [25]. As such, political factors influencing drug funding decisions may be complex and require further elucidation, and this was beyond the scope of our analysis.

### 4.4. Impact of Drug Class

We found statistically significant differences in TTF among drugs from different classes. Immunotherapy had the quickest TTF, while targeted therapies and chemotherapies took longer to receive funding approval. Immunotherapy is a relatively new and expanding area of cancer treatment, while chemotherapies have been used in the treatment of cancer since the early 20th century [26,27]. Therefore, there may be more pressure to approve and fund immunotherapies to expand this emerging treatment option. Relative efficacies and clinical benefits may play a role as well. In a systematic review, it was found that patients treated with immunotherapy were less likely to experience or die of treatment-related adverse events compared to those treated with chemotherapy [28]. Similarly, while there was no statistically significant difference in the decision to fund by agent class, numerically, a greater proportion of immunotherapy drugs were funded. The odds of funding chemotherapy drugs and targeted drugs were low in relation to those for immunotherapy drugs in logistic regression, although the sample size was too small to establish statistical significance. This may suggest a predisposition to fund novel agents such as immunotherapy.

### 4.5. Limitations

There are imitations with respect to this study. First, while we analyzed correlations among various factors and subsequent provincial funding decisions and timeliness, the study was not designed to assess causation, and therefore, definitive conclusions on the specific determinants of cancer drug funding cannot be drawn. While a multivariate analysis was conducted for the odds of funding drugs by agent class and list price, all four variables could not be included in the analysis due to inherent limitations in the statistical model. It was also not possible to conduct multivariate analysis for the TTF endpoint due to the small sample size. Second, we used list price to compare cost among the different drugs analyzed; however, provinces receive discounted drug prices following confidential negotiations with pCPA. Thus, the list price may not be an accurate reflection of the actual cost of the drugs. Third, in our calculations of TTF, we measured time to first provincial funding, which may not accurately reflect time delays across the remainder of the provinces. This method was selected to provide the most conservative time delay estimate, recognizing that the remainder of the provinces may take longer to fund or not fund at all. Lastly, our paper did not consider the impact of less tangible or measurable factors, such as the impact of “thought-leaders” for individual tumor types.

Finally, since these data were collected, the pCODR program has been amalgamated into the broader CADTH Reimbursement Review process.

## 5. Conclusions

In our analysis of 43 cancer drugs undergoing the funding decision process in Canada, we found that tumor type, drug class, and pCODR recommendation were determinants of the decision to fund and TTF by Canadian provinces. Meanwhile, drug price did not appear to play a significant role in funding decisions. There are many factors that may contribute to these findings, including drug clinical efficacy, cost effectiveness, and political pressure, but definitive conclusions are beyond the scope of this analysis. Given the relative importance of the pCODR recommendation in comparison to the list price, we may focus our efforts on expediting the submission to the HTA, the HTA process itself, and the funding approval process post HTA completion. Future studies should further investigate the role that these factors play in cancer drug funding decisions.

## Figures and Tables

**Table 1:** Drug descriptors.

Cancer Type	Drug Name	Indication	Agent Class	pCODR Final Recommendation	List Price * per 28-Day Course	TTF (Days)
Breast	Eribulin mesylate	Third line	Chemo	Fund, Conditional	CAD 4569.33	587
Breast	Everolimus	HR+, HER2/neu(−)	Targeted	Fund, Conditional	CAD 5208.00	302
Breast	Fulvestrant	HER2/neu(−)	Targeted	Fund, Conditional	CAD 1165.79	N/A
Breast	Lapatinib	HR+, HER2/neu+, in combination with letrozole	Targeted	Don’t fund	CAD 3948.00	N/A
Breast	Palbociclib	HR+, HER2/neu(−), in combination with letrozole	Targeted	Fund, Conditional	CAD 6250.00	698
Breast	Pertuzumab	HER2/neu+, in combination with trastuzumab and a taxane	Targeted	Fund, Conditional	CAD 8418.00	203
Breast	Trastuzumab emtansine	Second line, HER2/neu+	Targeted	Fund, Conditional	CAD 8426.88	202
Breast	Ribociclib	First line, HR+, HER2/neu(−)	Targeted	Fund, Conditional	CAD 6249.99	N/A
CRC	Aflibercept	Second+ line	Targeted	Don’t fund	CAD 2800.00	N/A
CRC	Bevacizumab	First line, with capecitabine	Targeted	Fund, Conditional	CAD 4200.00	4379
CRC	Cetuximab	First line, KRAS WT	Targeted	Don’t fund	CAD 6251.75	N/A
CRC	Panitumumab	First line, KRAS WT, with contraindication to bevacizumab	Targeted	Fund, Conditional	CAD 5174.06	702
CRC	Panitumumab	First line, KRAS WT, for left sided primary	Targeted	Do not fund	CAD 5391.29	N/A
CRC	Regorafenib	Third+ line	Targeted	Do not fund	CAD 6237.00	N/A
CRC	Trifluridine and tipiracil	Last line	Chemo	Do not fund	CAD 5631.00	N/A
Lung	Afatinib	First line, EGFR+	Targeted	Fund, Conditional	CAD 2240.00	291
Lung	Alectinib	First line	Targeted	Fund, Conditional	CAD 9446.08	245
Lung	Alectinib	Second line	Targeted	Fund, Conditional	CAD 9446.08	865
Lung	Alectinib	NSCLC with CNS metastases	Targeted	Do not fund	CAD 9452.80	N/A
Lung	Atezolizumab	Second line	I-O	Fund, Conditional	CAD 9034.67	311
Lung	Ceritinib	ALK+	Targeted	Fund, Conditional	CAD 9445.32	1210
Lung	Crizotinib	First line, ALK+	Targeted	Fund, Conditional	CAD 8213.34	1315
Lung	Crizotinib	Second line, ALK+	Targeted	Fund, Conditional	CAD 8213.34	524
Lung	Dabrafenib and trametinib	Second line, BRAF+	Targeted	Do not fund	CAD 15,669.70	N/A
Lung	Nivolumab	Second line	I-O	Fund, Conditional	CAD 8213.31	369
Lung	Osimertinib	First line, EGFR+	Targeted	Fund, Conditional	CAD 8250.94	N/A
Lung	Osimertinib	EGFR T790M mutation+	Targeted	Fund, Conditional	CAD 8250.94	818
Lung	Pembrolizumab	First line, PD-L1 ≥50%	I-O	Fund, Conditional	CAD 11,733.33	148
Lung	Pembrolizumab	Second+ line, PD-L1+	I-O	Fund, Conditional	CAD 8237.00	601
Lung	Pemetrexed	Maintenance following first line pemetrexed and cisplatin	Chemo	Fund, Conditional	CAD 5834.00	298
Melanoma	Cobimetinib	BRAF+	Targeted	Fund, Conditional	CAD 7567.00	465
Melanoma	Dabrafenib	BRAF+	Targeted	Fund, Conditional	CAD 7093.00	381
Melanoma	Dabrafenib and trametinib combo	BRAF+	Targeted	Fund, Conditional	CAD 15,213.64	503
Melanoma	Ipilimumab	First line,	I-O	Fund, Conditional	CAD 32,480.00	176
Melanoma	Ipilimumab	Second line	I-O	Fund, Conditional	CAD 32,480.00	151
Melanoma	Nivolumab	First+ line	I-O	Fund, Conditional	CAD 8213.31	523
Melanoma	Nivolumab and ipilimumab combo	First+ line	I-O	Fund, Conditional	CAD 34,305.23	N/A
Melanoma	Pembrolizumab	First+ line	I-O	Fund, Conditional	CAD 8213.34	367
Melanoma	Trametinib	BRAF+	Targeted	Fund, Conditional	CAD 8120.00	379
Melanoma	Vemurafenib	BRAF+	Targeted	Fund, Conditional	CAD 10,425.34	198
NET	Everolimus	NETs of GI/lung origin	Targeted	Fund, Conditional	CAD 5602.38	502
NET	Everolimus	NETs of pancreas origin	Targeted	Fund, Conditional	CAD 5208.00	0
NET	Sunitinib	NETs of pancreas origin	Targeted	Fund, Conditional	CAD 5304.79	0

Abbreviations: ALK, anaplastic lymphoma kinase; BRAF, v-raf murine sarcoma viral oncogene homolog B1; CNS, central nervous system; CRC, colorectal cancer; EGFR, epidermal growth factor receptor; GI, gastrointestinal; HER2/neu, human epidermal growth factor receptor 2; HR, hormone receptor; I-O, immunotherapy; KRAS, Kirsten rat sarcoma; NET, neuroendocrine tumor; NSCLC, non-small-cell lung cancer; PD-L1, programmed death-ligand 1; TTF time to funding; WT, wildtype. * Available publicly from: Reimbursement Review Reports | CADTH: Available online: https://cadth.ca/reimbursement-review-reports (accessed on 8 January 2021).

**Table 2:** Drug characteristics.

Feature	Category	Result (*n* = 43) *
Tumor Type (N, %)	Breast	8 (19%)
	Colorectal	7 (16%)
	Lung	15 (35%)
	Melanoma	3 (7%)
	Neuroendocrine	10 (23%)
Drug Class (N, %)	Chemotherapy	3 (7%)
	I-O	9 (21%)
	Targeted	31 (72%)
Drugs with a positive pCODR recommendation (N, %)		35 (81%)
Drugs funded in at least one province (N, %)		31 (72%)
Time to funding (median, IQR)		379 days (203–601)
List price per 28-day course (median, IQR)		CAD 8213 (5391–9445)

* Includes all drugs, irrespective of funding status, Abbreviations: I-O, immunotherapy.

**Table 3:** Time to funding based on drug characteristics.

Drug Characteristic (*n* = 31) *	Result	*p*-Value
Cost	Correlation coefficient	
List price	−0.20	0.28
Tumor Type	TTF (IQR)	
Breast	302 days (203–587)	
Colorectal	2541 days (702–4379)	
Lung	447 days (295–842)	
Melanoma	379 days (198–465)	
Neuroendocrine	0 days (0–502) ^†^	<0.001
Agent Class	TTF (IQR)	
Chemo	443 days (298–587)	
I-O	339 days (164–446)	
Targeted	465 days (245–702)	<0.01

* Includes only drugs that were funded in at least one province (*n* = 31), ^†^ Everolimus and sunitinib for pancreas NETs were funded in British Columbia before Health Canada approval (this is a highly unusual situation). Abbreviations: I-O, immunotherapy; TTF time to funding.

**Table 4:** Association of drug features and list price.

Feature	Category	List Price (Median, IQR)	*p*-Value
Tumor Type	Breast	CAD 5279 (CAD 4259–7334)	
	Colorectal	CAD 5391 (CAD 4200–6237)	
	Lung	CAD 8251 (CAD 8213–9446)	
	Melanoma	CAD 9319 (CAD 8210–32,480)	
	Neuroendocrine	CAD 5305 (CAD 5208–5602)	0.0004
Agent Class	Chemo	CAD 5631 (CAD 4569–5834)	
	I-O	CAD 9035 (CAD 8213–32,480)	
	Targeted	CAD 7093 (CAD 5208–8427)	0.0088
pCODR Recommendation	Fund	CAD 8213 (CAD 5602–9445)	
	Do not fund	CAD 5934 (CAD 4670–7852)	0.27
Funding Decision	Funded in at least one province	CAD 8213 (CAD 5602–9445)	
	Not funded	CAD 6244 (CAD 4670–8852)	0.34

## Data Availability

Data on drug details, approvals, and list prices: Reimbursement Review Reports; CADTH: Available online: https://cadth.ca/reimbursement-review-reports (accessed on 18 December 2021).
